# A Risk Assessment Comparison of Breast Cancer and Factors Affected to Risk Perception of Women in Turkey: A Cross-sectional Study

**Published:** 2017-03

**Authors:** Serpil YÜKSEL, Gülay ALTUN UĞRAŞ, İkbal ÇAVDAR, Atilla BOZDOĞAN, Sibel ÖZKAN GÜRDAL, Neriman AKYOLCU, Ecem ESENCAN, Gamze VAROL SARAÇOĞLU, Vahit ÖZMEN

**Affiliations:** 1. Dept. of Surgical Nursing, Division of Nursing, Faculty of Health Sciences, Necmettin Erbakan University, Konya, Turkey; 2. Dept. of Surgical Nursing, Division of Nursing, School of Health, Mersin University, Mersin, Turkey; 3. Dept. of Surgical Nursing, Florence Nightingale Faculty of Nursing, İstanbul University, İstanbul, Turkey; 4. Dept. of General Surgery, İstanbul Faculty of Medicine, İstanbul University, İstanbul, Turkey; 5. Dept. of General Surgery, Faculty of Medicine, Namık Kemal University, Tekirdağ, Turkey; 6. School of Medicine, Koç University, İstanbul, Turkey; 7. Dept. of Public Health, Faculty of Medicine, Namık Kemal University, Tekirdağ, Turkey

**Keywords:** Breast cancer, Gail risk model, Risk factors, Risk perception

## Abstract

**Background:**

The increase in breast cancer incidence has enhanced attention towards breast cancer risk. The aim of this study was to determine the risk of breast cancer and risk perception of women, factors that affect risk perception, and to determine differences between absolute risk and the perception of risk.

**Methods:**

This cross-sectional study was carried out among 346 women whose score in the Gail Risk Model (GRM) was ≥ 1.67% and/or had a 1^st^ degree relative with breast cancer in Bahçeşehir town in Istanbul, Turkey between Jul 2012 and Dec 2012. Data were collected through face-to-face interviews. The level of risk for breast cancer has been calculated using GRM and the Breast Cancer Risk Assessment Form (BCRAF). Breast cancer risk perception (BCRP), has been evaluated by visual analogue 100-cm-long scale.

**Results:**

Even though 39.6% of the women considered themselves as high-risk carriers, according to the GRM and the BCRAF, only 11.6% and 9.8% of women were in the “high risk” category, respectively. There was a positive significant correlation between the GRM and the BCRAF scores (*P*<0.001), and the BCRAF and BCRP scores (*P*<0.001). Factors related to high-risk perception were age (40–59 yr), post-menopausal phase, high-very high economic income level, existence of breast cancer in the family, having regular breast self-examination and clinical breast examination (*P*<0.05).

**Conclusion:**

In women with high risk of breast, cancer there is a significant difference between the women’s risk perception and their absolute risk level.

## Introduction

Breast cancer is one of the most frequent cancers around the world, also one of the most common causes of cancer-related death among women (1–5). It is responsible for almost 25% of all women’s cancers and about 12% of all new cancers (4). In 140 of 184 countries, breast cancer was declared the most common cancer (5). There will be a 50% increase in the rate and mortality of breast cancer between 2002 and 2020 due to demographic changes. Moreover, the rates of breast cancer and mortality due to breast cancer in developing countries in 2020 are expected to be 55% and 58%, respectively (2).

Breast cancer incidence, which is already high in developed countries, is also dramatically increasing in developing countries such as Turkey (2, 3, 6–10). The 2010 data of the Health Ministry of Turkey showed that breast cancer makes up 22.9% of all cancers in women (10), it is among the top 10 cancers in both women and men, with an incidence of 17.96/100000, and it was placed 4^th^ after lung, prostate, and skin cancer (8).

The rate of increase in breast cancer has also enhanced global breast health initiatives, and attention towards breast cancer risk and awareness (2, 6, 11–13). Breast cancer causes serious concerns even in healthy women, both because of its incidence and mortality. The steps that should be taken in order to decrease this threat can be arranged as following: assessment of breast cancer risk of women, determination of risk groups, careful monitoring of such high-risk groups, informing individuals with risk factors, and extending screening and reachable treatment programs in every society (2, 6, 7, 9, 11–17). Breast self-examination (BSE), mammography, clinical breast examination (CBE) are accepted as the most important screening methods in the early diagnosis of breast cancer (2, 3, 6, 12, 18–22). Effective application of screening programs depends on raising awareness of breast cancer and risk factors in women of aged more than 50 yr who are the target group of breast cancer (2, 3, 7, 12, 13, 23–25).

Breast cancer risk factors have been defined by previous studies. Age and female sex are important risk factors for breast cancer. Other factors can be increase breast cancer risk including personal and family history of breast, ovarian, and endometrium cancer; history of lobular carcinoma in situ-matched biopsy of atypical hyperplasia; positive BRCA 1 and BRCA 2 genes; early menarche (˂12 yr), late labor (˃30 yr); induced abortion; late menopause (˃55 yr); hormonal replacement treatment (HRT); alcohol over-consumption; smoking; lack of physical activity; diet rich in fat; body mass index (BMI); and high socio-economic level (1, 9, 11, 12, 14–17, 26).

The level of breast cancer risk can be calculated with risk models based on risk factors such as the Gail Risk Model (GRM), the Claus Model, the Tyrer-Cuzick Model, and the Breast Cancer Risk Assessment Form (BCRAF). Those risk models are essential to demonstrate the absolute risk, and thus to compare risk perception with actual risk (11–14, 16, 17, 21, 26, 27).

Determination of individual breast cancer risk perception (BCRP) is as important as the determination of the level of absolute risk (2, 6, 7, 12, 15, 16, 19, 24, 27–31). The perception of risk, health beliefs and attitudes, awareness in level of breast cancer, and risk factors affect the choice and application of early diagnostic screening methods (1, 2, 6, 7, 12, 18, 19, 23, 24, 29, 31). There are differences between the absolute risk level and risk level perception. Moreover, women who have low absolute risk consider themselves as being at high risk and have unnecessary fear (3, 6, 7, 9, 12, 19, 24, 27–31). Contrary to this, women with low perception of risk may not care about early diagnostic methods such as BSE, CBE, and mammography (3, 6, 12).

The aim of this study was to determine the risk of breast cancer and risk perception of women, factors that affect risk perception and to determine differences between absolute risk and the perception of risk. In addition, we aimed to show a relation between BCRAF and GRM.

## Materials and Methods

### Design

This study was designed as a cross-sectional study based on society.

### Participants and setting

This study was conducted in Bahçeşehir town in Istanbul, which is the most populous and most important city from economic and socio-cultural aspects in Turkey’s northwest. Bahçeşehir town was Turkey’s first satellite city, an important commercial and business center. In 2008, the Turkey Breast Health Association launched the Bahçeşehir Community-Based Breast Cancer Screening Program in this town, which has a quality population and residence register system. The program that provided free of charge for all services, is the first organized population-based breast cancer screening program in Turkey. Overall, 5680 women aged 40–69 yr and only live in the Bahçeşehir town were attended in the program, planned to last 10 yr (2008–2018). Screening activities were initiated at the end of 2008. The population for this study consisted of 5530 women aged between 40–69 yr who live in the Bahçeşehir town and attended the Bahçeşehir Community-Based Breast Cancer Screening Program between Jan 2009, and Jun 2012. Among these women, 425 women were selected as samples to the study program because their GRM score was ≥1.67% and/or they had a 1^st^ degree relative with breast cancer. The participants had not been diagnosed as having breast cancer. Three hundred eighty of these 425 high-risk women were accepted to participate in the study. Twenty-four women who did not respond to all of the questions and 10 who wished to leave the study were excluded from the final analysis. The research was completed with 346 women, 81.4% of the study sample.

Tables published by WHO were used to determine the sample size of the study. A 17.6% rate of breast cancer risk of Turkish women (17) was assumed, and to estimate the real value of this ratio (0.04 points with 95% confidence), the required sample size was found to be 306 (32). The study was completed with a larger sample (n=346 women) than this value.

### Data collecting tools

The data collection tools included a questionnaire form, Gail Risk Assessment tool, BCRAF and a visual analog scale for breast cancer risk perception.

### Questionnaire form

Data related to descriptive features and breast cancer risk factors of women were collected with the questionnaire form. This form was conceived through a review of published study (3, 6, 7, 11–14, 16, 17, 19, 21, 24–27, 29, 30), and comprised two sections. The first section consisted of seven questions about descriptive features such as age, marital status, economic status, and education. The second section consisted of twenty questions on breast cancer risk factors such as age of menarche, family history of breast cancer, and history of breast biopsy. The comprehensibility of the questionnaire for participants was checked by two Turkish Language Experts and was then tested with a group of women (n=50) outside the study sample in a pilot study. The results of the pilot study were determined, and no changes were made to the questionnaire.

### Gail Risk Assessment Tool

The Gail Risk Assessment tool, also known as the Breast Cancer Risk Assessment tool, is one of the most commonly used models (6, 9, 17, 26, 28). GRM was developed and its validity has been tested in large populations (14). It is only used in women aged 35 yr or more and cannot be applied to those with a history of breast cancer and lobular or ductal carcinoma *in situ* (17, 33). This model calculates a woman’s risk of developing breast cancer within the next 5 yr and within her lifetime by using individual risk factors. These factors are age, menarche age, age of first live birth or no birth, the number of first-degree relatives who have had breast cancer, number of breast biopsies, breast biopsy with atypical hyperplasia, and race/ethnicity (6, 9, 12–14, 17, 24, 29, 31, 33). Women with 5-year GRM scores ≥1.67% are accepted as “at risk” (6, 9, 13, 14, 17, 29, 31). In some studies, the lifetime breast cancer risk according to GRM was classified as “usual = low (<15%),” “average = moderate (15–30%),” or “strong = high (>30%)” (7, 27). In this study, the GRM score of women was calculated with a computer program developed by scientists at the National Cancer Institute and National Surgical Adjuvant Breast and Bowel Project by the researchers (33) and their calculated risks were classification as low, moderate, and high.

### Breast Cancer Risk Assessment Form (BCRAF)

BCRAF, developed by American Cancer Society (ACS), is also accepted and recommended by the Turkish Ministry of Health to be used to determine the level of breast cancer risk. This form consists of 6 sections and 20 items. The objectives in the form, used to determine the level of breast cancer risk, include age, family history, personal history, birth giving age, menstrual history, and women’s body structure. Each answer for the related risk factor is graded by assigning points and a total score is calculated to determine the risk level. Breast cancer risk according to the total risk score is classified as low risk (200 points and below), moderate risk (201 to 300 points), high risk (301–400 points), and the highest risk (more than 400 points) (11, 16, 26).

### Visual analogue scale for breast cancer risk perception

BCRP was evaluated using a 100-cm-long visual analogue scale. Level of risk perception was determined using the results taken from the scale and defined as: 0% no risk, 1%–49% low risk, 50% moderate risk, 51%–100% high risk (1, 3, 27).

### Data collection

Participants were visited in their houses. The data of this study was collected between Jul 2012 and Dec 2012 through face-to-face interviews with the participants. The interviews lasted approximately 20 min.

### Ethical approach

Ethical approval was obtained before the study began from the Local Ethics Committee of University of Istanbul, Istanbul Faculty of Medicine (No: 04). The participants were informed that their participation was based on voluntariness.

### Data analysis

Full data were collected from 346 women who were all included in the final analysis. Data were analyzed using frequency, percentage, mean, standard deviation, Pearson’s Chi-square test, Spearman’s correlation, and logistics regression analysis. The relationship between risk assessment models and BCRAF score was evaluated with Spearman’s correlation (*P*<0.001 level, 99.9% confidence intervals (CI)). Whether there was a difference among the BCRP and descriptive features and protection behaviors of breast cancer was evaluated using Pearson’s Chi-square test (*P*<0.05 level, 95% CI). In addition, logistic regression analysis was used to identify factors that affected the BCRP level (*P*<0.05 level, 95% CI).

## Results

### Women’s characteristics and breast cancer risk factors

Totally, 346 women participated in the study. Women’s average age was 58.04 ± 8.25 yr (range, 40–69 yr; data not shown); most were married (76.6%) and in their post-menopausal phase (73.1%); according to their BMI, 40.5% were overweight and 31.8% were obese. About 63% of the women had menarche after the age of 12 yr; 93.6% had had live births; 72.8% had had their first birth before the age of 30 yr, and 91.4% had breastfed their babies; 24.3% had a history of breast-related disease; 23.1% had undergone biopsy; and among those who had a history of breast cancer in their family (58.7%), 87.7% had a history of breast cancer in their 1^st^ degree relatives (Table 1).

**Table 1:** Descriptive features and breast cancer risk factors

**Age groups (n=346)**	**n (%)**
40–59	176 (50.9)
60–69	170 (49.1)
Marital status (n=346)	
Single	81 (23.4)
Married	265 (76.6)
Educational status (n=346)	
Literate and primary school	131 (37.9)
Secondary-high school and higher	215 (62.1)
Economic status (n=346)	
Low-middle	143 (41.3)
High-very high	203 (58.7)
Body Mass Index (n=346)	
Normal (18.5–24.99)	96 (27.7)
Overweight (25.0–29.99)	140 (40.5)
Obese (≥30)	110 (31.8)
Menarche age (n=346)	
≤ 12 yr old	128 (37.0)
> 12 yr old	218 (63.0)
Live birth (n=346)	
Yes	324 (93.6)
No	22 (6.4)
Age of first birth (n=324)	
<30 yr old	236 (72.8)
≥30 yr old	88 (27.2)
Breast feeding (n=324)	
Yes	296 (91.4)
No	28 (8.6)
Menopausal status (n=346)	
Premenopausal	93 (26.9)
Postmenopausal	253 (73.1)
Having hormone replacement therapy after menopause (n=253)	
Yes	72 (28.5)
No	181(71.5)
History of breast related diseases (n=346)	
Yes (Benign mass, cyst, apses)	84 (24.3)
No	262 (75.7)
History of breast biopsy (n=346)	
Yes	80 (23.1)
No	266 (76.9)
Family history of breast cancer (n=346)	
Yes	203 (58.7)
No	143 (41.3)
Relative degree of breast cancer patient (n=203)	
1^st^ degree relative	178 (87.7)
2^nd^ degree relative	25 (12.3)

### Breast cancer risk, risk perception and the correlation between risk models

Some 39.6% of women had high scores in the BCRP scoring system; according to the GRM and BCRAF scores, only 11.6% and 9.8% were in the high-risk category, respectively (Table 2). There was a positive significant correlation between the GRM and BCRAF scores (r=0.472, *P*<0.001), and the BCRAF and BCRP scores (r=0.294, *P*<0.001) (Table 3).

**Table 2:** Breast cancer risk assessment

**Breast cancer risk profile**	**n**	**%**
BCRP Score (n=346)		
No Risk (0 points)	33	9.5
Low Risk (1–49 points)	132	38.2
Moderate Risk (50 points)	44	12.7
High Risk (51–100 points)	137	39.6
BCRAF Score (n=346)		
Low Risk (200 points)	90	26.0
Moderate Risk (201–300 points)	222	64.2
High Risk (301–400 points)	34	9.8
GRM Score (n=346)		
Low Risk (< 1.67)	46	13.3
Moderate Risk (1.67–2.99)	260	75.1
High Risk (≥ 3)	40	11.6

**Table 3:** Relation between risk assessment models

**Risk assessment models**	**BCRP Score**	**BCRAF Score**
BCRAF Score	r = 0.294*	
GRM Score	r = 0.074**	r = 0.472*

* *P*<0.001;

** *P* > 0.05;

r=Spearman Correlation

However, there was no significant correlation between the GRM and BCRP scores (r=0.074, *P*>0.05) (Table 3). BCRP level had significant differences according to age, menopausal situation, economic situation, family history of breast cancer, practice of BSE, and having regular CBE (*P*˂0.05, Table 4).

**Table 4:** Relation between risk perception and descriptive features and protection behaviors of breast cancer

**Features**		**BCRP**				
		**Low-Moderate**		**High**		***P***
		**n**	**%**	**n**	**%**	
Age	40 – 59 (yr)	89	42.6	87	63.5	0.001
	≥ 60 (yr)	120	57.4	50	36.5	
Menopausal status	Premenopausal	43	20.6	50	36.5	0.001
	Postmenopausal	166	79.4	87	63.5	
Marital status	Married	153	73.2	112	81.8	0.066
	Single	56	26.8	25	18.2	
Educational status	Literate and Primary School	84	40.2	47	34.3	0.270
	Secondary-High School and Higher	125	59.8	90	65.7	
Economic status	Low-Middle	101	48.3	42	30.7	0.001
	High-very high	108	51.7	95	69.3	
Work status	Not working	182	87.1	113	82.5	0.305
	Working	27	12.9	24	17.5	
BMI	Normal	53	25.4	43	31.4	0.221
	Overweight - obese	156	74.6	94	68.6	
Age of menarche	≤ 12 yr old	77	36.8	51	37.2	0.942
	> 12 yr old	132	63.2	86	62.8	
Live birth	Yes	194	92.8	130	94.9	0.506
	No	15	7.2	7	5.1	
Miscarriage	Yes	45	21.5	30	21.9	0.935
	No	164	78.5	107	78.1	
Abortion	Yes	109	52.2	79	57.7	0.314
	No	100	47.8	58	42.3	
Breast feeding	Yes	183	87.6	113	82.5	0.247
	No	26	12.4	24	17.5	
HRT	Yes	44	26.5	28	32.2	0.342
	No	122	73.5	59	67.8	
Breast related diseases	Yes	51	24.4	33	24.1	0.947
	No	158	75.6	104	75.9	
History of breast biopsy	Had	45	21.5	35	25.5	0.386
	Did not have	164	78.5	102	74.5	
Family history of breast cancer	Yes	93	44.5	110	80.3	<0.001
	No	116	55.5	27	19.7	
Healthy nutrition	Yes	141	67.5	101	73.7	0.214
	No	68	32.5	36	26.3	
Making regular exercises	Yes	77	36.8	43	31.4	0.294
	No	132	63.2	94	68.6	
Practice of BSE	Yes	150	71.8	121	88.3	<0.001
	No	59	28.2	16	11.7	
Having regular CBE	Yes	144	68.9	118	86.1	<0.001
	No	65	31.1	19	13.9	
Mammogram periods (year)	≤ 1 year	72	34.4	56	40.9	0.226
	> 1 year	137	65.6	81	59.1	

Higher BCRP levels were found in women aged between 40–59 yr, women in the post-menopausal phase, women whose economic situation was in the high to very high level, women with a history of breast cancer in their families, and women who had regular BSE and CBE (*P*˂0.05, Table 4). In the logistics regression analysis, independent factors related to high-risk perception were high-very high economic income level, existence of breast cancer in the family, practice of BSE, and regular CBE (Table 5).

**Table 5:** Factors that affect breast cancer risk perception level

	**Factors**	**OR**	**95% Confidence Interval**	***P***
Model 4*	High-very high economic income level	2.074	1.243–3.460	0.005
	Family history of breast cancer	5.542	3.267–9.403	0.001
	Practice of BSE	2.454	1.269–4.745	0.008
	Having regular CBE	2.751	1.488–5.084	0.001
	Constant	0.273	−	0.000

* Logistics regression analysis: Logistic Regression (Method = Forward Stepwise)

Dependent Variable: Breast Cancer Risk Perception (1 = High; 0 = Low/moderate)

## Discussion

Breast cancer is an important public health problem, which has worldwide increasing prevalence (2–4, 6, 13). Although it is not possible to prevent breast cancer with today’s medical facilities, early diagnosis has vital importance (2, 3, 8, 12, 16, 17, 23). Determination of breast cancer risk increases the chance of early diagnosis (3, 9, 14). The aim of this study was to determine the breast cancer risk and risk perception of women, factors that affect risk perception, differences between the absolute risk and perception of risk, and relationship between BCRAF and GRM.

### Breast cancer risk perception

Studies that focused on the relationship between BCRP and use of early diagnostic methods have demonstrated that risk perception affects the application of early diagnostic methods (3, 7, 18, 19, 21, 23–25). Perception of high risk increased the frequency of BSE (18, 19, 25), mammography (3, 7, 18, 21, 23, 25), and CBE (3). On the other hand, there was no relationship between risk perception and the frequency of performing early diagnostic methods such as BSE (3, 6, 7, 12, 28), CBE (12, 20, 25, 28), and mammography (6, 9, 20, 22, 24, 28). Family history of breast cancer affects the application of early diagnostic methods (17, 18, 22, 23). Women who had a family history of breast cancer, especially a 1^st^ degree relative with breast cancer, were tended to undergo significantly more mammography scans (17, 18, 22, 23), CBE (17), and BSE (18). There was a significant correlation between family history of breast cancer and repetition of mammography (22). A history of breast cancer in family significantly affects risk perception, and that risk perception significantly affects the performance BSE, and demand for CBE, which supports previous studies in which positive relationships were found, yet disagrees with statements of insignificant correlation. In a similar manner, while data given on irrelevancy of BCRP and mammography requests from women upholds previous results that perception of high risk does not increase the demand for or frequency of mammography; it also disagrees with studies that revealed a positive relationship. The reason why high-risk perception was not related to the frequency of mammography may have been due to the regular intervals of mammographic screening as a part of the Bahçeşehir Community-Based Breast Cancer Screening Program.

Age and/or breast cancer cases in a family were significantly associated with breast cancer risk perception (1, 3, 6, 7, 9, 12, 15, 18, 22, 23, 28–31). Age, family history of breast cancer, and higher education level were factors that were significantly associated with higher perceived risk of breast cancer (15). There was a positive correlation between risk perception and breastfeeding, history of breast disease, and family history of breast cancer; however, there was no such relationship between breast biopsy, menopausal phase, and perception of risk (12). Women aged between 45–54 yr who had an education level equal to a university degree and who worked, perceived their risk as “high,” more than women in older age groups (6). The accuracy of breast cancer risk estimation was not affected by the presence of a first-degree relative with breast cancer. In a similar study that was conducted with women whose sisters had breast cancer, participants aged younger than 50 yr had a lifelong high-risk perception (30). Educational level, marital status, working status, and breast cancer in the family did not influence the accurate perception of breast cancer risk, while high-income level, aged 50–59 yr, and being in the post-menopausal period did influence this perception (9). The results of our study support data in the literature by defining a significant interaction between risk perception and age, family history, breast biopsy, and economic status. In addition, our results also support the literature through demonstrating that risk perception was higher in women who were in the menopausal phase, aged 40–59 yr, and conducting regular BSE. Further, our data states that risk perception was not affected by level either of education or by working status or by marital status. Contrary to the literature, the effects of BMI, personal breast disease history, and breastfeeding were negligible on risk perception, which also differs from other results in literature. The Bahçeşehir Community-Based Breast Cancer Screening Program has increased awareness in its participants about early diagnostic methods. In addition, unmodifiable risk factors such as high BMI, breastfeeding, and breast disease history do not affect women’s risk perception.

### Risk perception and absolute risk in breast cancer

Risk perception of breast cancer among women is higher than their absolute risk (3, 6, 7, 9, 24, 28, 31). There was a significant difference between perceived and absolute risks (7); 28.4% of participants defined themselves as being at moderate risk and 7.4% thought that they belonged in the high-risk group, whereas the percentages of absolute moderate and high-risk groups were 0.9% and 0%, respectively, according to the GRM score. Among women aged more than 50 yr, the average BCRP scores were greater than GRM scores (28). 44.4% of women had a BCRP score of 50% and higher (3). 47.8%–81% of women believed that their risk was higher than it was in reality (15, 24). Similarly, 72% of moderate risk and only 18% of high-risk women determined their risk levels accurately, which appoints that age is important in assessing risk (31). Complementary and parallel results to the literature were also obtained in our study. Table 2 depicts that 39.6% of participants had a high level of breast cancer risk perception; however, only 11.6% and 9.8% were in the high-risk category according to the GRM and BCRAF, respectively. According to the BCRAF and GRM results in our study, 64.2% and 75.1% of women were in the moderate risk category, respectively, and 12.7% were in moderate risk category according to BCRP score. These findings are important because similar risk assessments are performed using BCRAF and GRM. The positive significant relation between GRM and BCRAF scores findings also supports this result. Despite the risk being calculated as low, the reason for the perception of high risk may be due to 58.7% of the attendants having had breast cancer in their family.

### Limitations

There are some limitations to the present study. The study included only a small population of women at high risk of breast cancer who lived in a particular area and were registered with the Bahçeşehir Community-Based Breast Cancer Screening Program. Consequently, the ability to generalize data from this study for the Turkish female population is limited. The reasons behind the difference of perceived and actual risk are not explained extensively in this study. Moreover, the level of breast cancer risk awareness is not displayed.

## Conclusion

There was a significant difference between BCRP and absolute risk. In addition, a positive significant correlation was between the GRM and BCRAF scores, and the BCRAF and BCRP scores. Independent factors related to high-risk perception were high to very high economic income level, existence of breast cancer in the family, practice of BSE, and having regular CBE.

## Ethical considerations

Ethical issues (Including plagiarism, informed consent, misconduct, data fabrication and/or falsification, double publication and/or submission, redundancy, etc.) have been completely observed by the authors.
